# Synthesis, *in vitro* aerobic and hypoxic cytotoxicity and radiosensitizing activity of novel metronidazole tethered 5-fluorouracil

**DOI:** 10.1186/2008-2231-21-76

**Published:** 2013-12-20

**Authors:** Khosrou Abdi, Ali Khalaj, Seyed Nasser Ostad, Navid Lamei, Mohammad Reza Khoshayand

**Affiliations:** 1 Department Radiopharmacy, Tehran University of Medical Sciences, Tehran 14174, Iran; 2 Department of Medicinal Chemistry, Tehran University of Medical Sciences, Tehran 14174, Iran; 3 Department of Toxicology & Pharmacology, Tehran University of Medical Sciences, Tehran 14174, Iran; 4 Drug Design & Development Research Center, Tehran University of Medical Sciences, Tehran 14174, Iran; 5 Department of Drug and Food Control, Faculty of Pharmacy, Tehran University of Medical Sciences, Tehran 14174, Iran

**Keywords:** Cytotoxicity, Radiosensitizing, Metronidazole, 5-Fluorouracil, MTT& PI-digitonin assays

## Abstract

**Background and the purpose of the study:**

Several 2, 4-dinitrophenyl and 2,4-dinitrophenylamine tethered 5-FU (5-fluorouracil) compared to their components have shown minimal or no cytotoxicity to HT-29 cell line under aerobic conditions but high cytotoxicity and radiosensitizing effects under hypoxic conditions. In the present study the cytotoxicity and radiation potentiation of three novel analogues of these compounds by replacing 2,4-dinitrophenyl moiety with 2-methyl-5-nitroimidazole, a known radiosensitizer and cytotoxic agent was investigated.

**Methods:**

Tethered compounds **7**–**9** were prepared by the reaction of 1-(*t*-butoxycarbonyl)-5-fluorouracil **6** with metronidazole esters **2**–**4** followed by removal of the *t*-butoxycarbonyl protecting group. Cytotoxicity of compounds in HT-29 cells with or without radiation were determined by 3-(4,5-Dimethylthiazol-2-yl)-2,5-diphenyltetrazolium bromide (MTT), propidium iodide (PI)-digitonin and clonogenic assays.

**Results:**

Tethered compounds **7**–**9** induced time-and concentration–dependent cytotoxicity under hypoxia but had no significant effect under aerobic conditions. These compounds also showed selective and concentration- dependent radiosensitization effects under hypoxic conditions.

**Conclusion:**

Tethered compounds **7**–**9** compared to 5-FU **5** showed minimal cytotoxicities under aerobic and selective radiosensitizing activities under hypoxic conditions. Also effects of these compounds were higher than those of metronidazole **1** which is a known cytotoxin and radiosensitizer under hypoxic conditions.

## Introduction

Oxygen as a radiosensitizer increases the effects of radiation by the formation of free radicals that damage DNA [1]. Electron affinic nitroaromatic and nitroheterocyclic compounds act like oxygen by formation of nitro radical anion through one electron reduction and are solid tumor targeting agents because of the presence of hypoxic cells in these tumors due to insufficient blood supply [2]. The cytotoxicity of these compounds under hypoxia has also been attributed to an increase in the oxidative damage of DNA, formation of hydroxyl compounds under deoxygenated conditions, and suppression of intracellular radioprotectors of sulfhydryl compounds [3]. Under aerobic conditions where the initially reduced species can be readily re-oxidized by molecular oxygen, the cytotoxicity of these compounds has been attributed to the parent nitro compounds, or bio-activation by NADPH: quinine oxidoreductase (DT diaphorase) which can act as an oxygen insensitive reductase [4].

Nitroimidazoles [1,2] specifically 2- and 5-nitro derivatives are known as cytotoxin and radiosensitizer toward hypoxic cells and have been subject of many clinical studies. However these compounds have shown limited therapeutic impact on radiotherapy due to clinical problems that includes dose-limiting side effects such as neurotoxicity [5]. Additionally several hybrid or dual functional agents have been described in which nitroimidazoles are attached through a linker unit to cytotoxic nitrogen mustards, [1,2,6] and aziridine moieties [1,2,7] which have shown higher cytotoxicity and enhanced radiosensitizing activity than those of the corresponding counterparts in both aerobic and hypoxic cells. In these compounds cellular reduction of the nitro group to more electron donating species activate the nitrogen of the cytotoxic group by the release of electron through the aromatic ring and generate reactive alkylating species [7].

Previously, results of our study on *in vitro* aerobic cytotoxicity of 2, 4-dinitrophenylamine tethered 5-FU through three carbon atoms in HT-29 cell line with and without radiation showed that the compound unlike its components has no cytotoxicity but is radiosensitizer [8]. Since in the tested compound 5-FU is not a leaving group, it was reasoned that the aerobic cytotoxicity of the reported 2,4-dinitrophenylamine mustard [6] and 2,4-dinitrophenyl aziridine [7] are due to their alkylating moieties.

Also results of our investigations on 2,4-dinitrobenzyl, 2,4-dinitrobenzoyl, 3,5-dinitrobenzoyl and 2,4-dinitrophenacetyl derivatives of 5-FU showed that with exception of 3,5-dinitrobenzoyl derivatives other compounds have selective- cytotoxicity and radiosensitization effects under hypoxic conditions [9]. Since the IC_50_ values of compounds which showed selective cytotoxicity were not different significantly it was concluded that they have similar mechanism of action and might produce the same reactive species [9].

In continuation of our investigations on this type of bifunctional compounds [8-11] in the present study the cytotoxicity and radiosensitization effects of three novel analogues of these compounds by replacing 2,4-dinitrophenyl moiety with 2-methyl-5-nitroimidazole (metronidazole), a known radiosensitizer and cytotoxic agent [2,12] was investigated. This manuscript describes cytotoxicities and radiosensitizing activities of metronidazole tethered 5-FU through ethyl and labile ester groups which was hypothesized upon *in vivo* hydrolysis to generate active metabolites which might have synergistic actions.

## Materials and methods

### Chemistry

Metronidazole **1**, 5-FU **5**, di-*t*-butoxydicarbonate (Boc)_2_O, triethylamine, sodium hydride and other chemicals and solvents not listed here were from Merck Chemical Co. (Merck, Germany). Thin layer chromatography was carried out using Kieselge l60, 230–400 mesh, (Merck, Germany) to monitor the progress of reactions. Melting points were determined on a MEL-270 Sibata melting point apparatus and are uncorrected. ^1^HNMR spectra were recorded on 500 MHz a Bruker (Germany), using DMSO-d_6_ or CDCl_3_ as solvent. Chemical shifts (δ) are reported in ppm relative to TMS as internal standard. Mass spectra were obtained on a Finningan TSQ-70 (USA) instrument. Infrared spectra were recorded on a Nicolet Magna IR-550 (USA) spectrometer. All compounds were characterized by the above techniques. Analytical and spectroscopic data for the known compounds were consistent with the reported literature values and data only for new compounds are presented.

#### Synthesis of 2-(2-Methyl-5-nitro-1H-imidazole-1-yl) ethyl-2-chloroacetate (3)

To a solution of metronidazole **1** (3.9 g, 24 mmol) in 100 ml of anhydrous dichloromethane was added 1.9 ml (24 mmol) triethylamine at temperature below 5°C and after stirring for 30 min the solution was treated with 3.2 ml (40 mmol) of chloroacetyl chloride dropwise within 5 min and then the resulting solution was stirred at room temperature overnight. The mixture was filtered and the residue after evaporation of the solvent was subjected to flash chromatography using Pet-ether: EtOAC as eluting solvent (80:20) to give 7.9 g (64%) of the compound **3**. mp 80-82°C; ^1^H NMR (DMSO-d_6_): δ 8.01 (s, 1H-imidazole), 4.61 (t, *J* = 5.12 Hz, 2H, CH_2_-O), 4.48 (t, *J* = 5.12Hz, 2H, CH_2_-N), 4.35 (s, 2H, CH_2_-Cl), 2.18 (s, 3H, CH_3_); FT-IR (KBr): 1720 (C = O), 1546, 1350 (NO_2_) cm^-1^.

#### Synthesis of 2-(2-Methyl-5-nitro-1H-imidazole-1-yl)ethyl-3-chloropropanoate (4)

The reaction of metronidazole **1** (3.9 g, 24 mmol) with 3.85 ml (40 mmol) of 3-chloropropionyl chloride under above conditions for the preparation of the ester **3** but purification of the product by crystallization from Pet-ether: EtOAC instead of flash chromatography gave 4.1 g (71%) of the ester **4**. mp 89-93°C;^1^H NMR (DMSO-d_6_): δ 8.01 (s, 1H-imidazole), 4.58 (t, *J* = 5.10 Hz, 2H, CH_2_-O), 4.40 (t, *J* = 5.10 Hz, 2H, CH_2_-N), 3.7 (t, *J* = 5.3 Hz, 2H, CH_2_-Cl), 2.5 (t, *J* = 5.3 Hz, 2H, CH_2_-C = O), 2.18 (s, 3H, CH_3_); FT-IR (KBr): 1729 (C = O), 1540, 1345 (NO_2_) cm^-1^.

#### General procedure for the synthesis of tethered compounds 7–9

To a stirred solution of 225 mg (1 mmol) of 1-(*t*-butoxycarbonyl)-5-FU **6**[13] in 10 ml of anhydrous DMF in a round bottom flask which was kept in an ice bath was added 30 mg (1.25 mmol) of sodium hydride within 10 min and after stirring for 30 min at 5°C the mixture was then treated with a solution of 1 mmol of metronidazole esters **2-4** in 10 ml of anhydrous DMF and the mixture stirred at room temperature for 18 h. The mixture was then diluted with the same volume of water, extracted with ethyl acetate (3×20 ml), and the organic layer was dehydrated using anhydrous sodium sulfate. Evaporation of ethyl acetate gave an oily residue which was dissolved in 10 ml of methanol, treated with 0.42 g potassium carbonate and the obtained mixture was stirred at room temperature for 1 h. After evaporation of the solvent under reduced pressure, the residue was dissolved in water-ethyl acetate mixture (1:1), the organic layer was separated and dehydrated using anhydrous sodium sulfate and then evaporated under reduced pressure. For the preparation of compound **7**, the residue after evaporation of the solvent was purified by flash chromatography using Pet-ether: EtOAC as eluting solvent (70:30) to give 100 mg (35%) of the product. For the preparation of compounds **8** and **9**, residues were crystallized from Hexan-EtOAC to give 310 mg (92%) and 330 mg (93%) of the pure products respectively.

#### 5-fluoro-3-(2-(2-methyl-5-nitro-1H-imidazol-1-yl)ethyl)pyrimidine-2,4(1H,3H)-dione(7)

mp 185-189°C; ^1^HNMR (CDCl_3_): δ 7.9 (s, 1H-imidazol), 7.8 (d, 1H, CH = CF), 4.0 (t, CH_2_), 3.9 (t, CH_2_), 2.4 (s, 3H, CH_3_); FT-IR (KBr): 3108 (NH), 1722, 1671 (C = O), 1550, 1350 (NO_2_) cm^-1^; MS: m/z 81(100%), 130 (78%), 238 (39%), 155(24%).

#### 2-(2-methyl-5-nitro-1H-imidazol-1-yl)ethyl 2-(5-fluoro-2,6-dioxo-2,3-dihydropyrimidin-1(6H)-yl)-acetate(8)

mp 231–234°C; ^1^H NMR (DMSO-d_6_): δ 7.99 (s, 1H-imidazole), 7.93 (d, *J* = 8.5 Hz, 1H, CH = CF), 4.65 (t, *J* = 5.02 Hz, 2H, CH_2_-O), 4.42 (s, 2H, CH_2_-C = O), 3.98 (t, *J* = 5.02 Hz, 2H, CH_2_-N), 2.42 (s, CH_3_); FT-IR (KBr): 3131 (NH), 1725, 1656 (C = O), 1542, 1349 (NO_2_) cm^-1^; MS: m/z 341 (7%), 296 (23%), 212 (80%), 171(74%), 130 (100%), 126 (42%).

#### 2-(2-methyl-5-nitro-1H-imidazol-1-yl) ethyl 2-(5-fluoro-2,6-dioxo-2,3-dihydropyrimidin-1(6H)-yl)-propanoate(9)

mp 247-250°C; ^1^H NMR (DMSO-d_6_): δ 8.01 (s, 1H-imidazole), 7.98 (d, *J* = 8.5Hz, 1H, CH = CF), 4.57 (t, *J* = 5.05 Hz, 2H, CH_2_-O), 4.39 (t, *J* = 5.05 Hz, 2H, CH_2_-N), 3.78 (t, *J* = 6.8 Hz, 2H, CH_2_-N), 2.70 (t, *J* = 6.8 Hz, 2H, CH_2_-C = O), 2.44 (s, CH_3_); FT-IR (KBr): 3124 (NH), 1721, 1652 (C = O), 1547, 1350 (NO_2_) cm^-1^; MS: m/z 355 (5%), 309 (21%), 226 (86%), 185(75%), 171 (42%), 130 (100%), 126 (59%).

#### In vitro cytotoxicity

##### Materials

HT-29 cells, originally derived from human colorectal carcinoma were obtained from Pasteur Institute (Tehran, Iran). Fetal Bovine Serum (FBS), Penicillin/Streptomycin 100X, Trypsin-EDTA 10X and Trypan blue were from Biosera (England). RPMI 1640 from Gibco (England), MTT and PI from Sigma (USA) and Digitonin from Merck (Germany) were used in this study.

##### Cell culture

HT-29 cells were grown as an attached monolayer in RPMI 1640 supplemented with 10% fetal bovine serum, penicillin (50 unit/ml), and streptomycin (50 μg/ml). Cells were routinely grown in tissue culture flasks and kept in a humidified atmosphere of 5% CO_2_ and 95% air at 37°C [14].

### Methods of assay

Cytotoxicity of 5-FU**5**, metronidazole **1**, and tethered compounds **7**–**9** against HT-29 cells under both aerobic and hypoxic conditions were determined by measurement of the percentage of cell survivals using MTT [15] and PI-digitonin [16,17] and clonogenic forming assays [18]. Cells were seeded at the density of 10^4^ cells/well in 96-well culture dishes and incubated for 4 h. Stock solutions of the tested compounds in DMSO at concentrations of 50 mmol which were prepared prior to the experiments diluted with culture media and at concentrations of 6.25-400 μmol/ml added to each well of plates. Cells in the control wells were treated with the same volume of medium which contained DMSO at concentration equal to those which were used in incubations treated with the tested compounds. Cell incubations were kept at 37°C under either air by purging with 95% nitrogen and 5% CO_2_ or hypoxia in sealed GasPack jar by using Anoximat (Netherland) and an anaerobic chamber GasPak Jar (Merck, Germany) and GasPak pluss (Merck, Germany) [19] for 3 or 48 h. After removal of the supernatant of the culture medium, wells were washed with phosphate buffer saline (PBS), and the numbers of live cells were determined by following methods.

#### MTT assay

25 μl of the reagent (5 mg/ml in PBS) was added to each well, cell cultures were incubated for 4 h at 37°C and then the precipitated formazan was dissolved in 100 μl of DMSO and its absorbance was read in Elisa reader at 550 nm and a background wavelength of 690 nm. Survival was scored by the ratio of the absorbance of the treated cells to untreated cells with the tested compounds (control) and is expressed as percentage of the cell survival [15].

#### PI-digitonine assay

20 μl of a 1.53 mmol solution of PI in PBS for a final concentration of 30 μmol was added to each well, cell cultures were incubated for 60 min at 37°C and then the initial fluorescence intensity from the dead cells was measured in a multi-well plate reader, (BioTek, Synergy HT, USA) with 530-nm excitation and 645-nm emission filters. Then digitonin (600 nmol) was added to each well to permeabilize all cells and label all nuclei with PI and after 30 min incubation, the fluorescence intensity was measured again to obtain a value corresponding to the total number of cells. The percentage of dead cells was calculated as the proportion of fluorescence intensity of the dead cells to that of total cells multiplied by 100 and percentage of the cell viability was calculated by subtracting the percentage of dead cells from 100% [16].

The IC_50_ values for both MTT and PI-digitonine assays were determined from cell survival curves in which the percentages of cells that survived were plotted as a function of the concentration of the tested compounds.

#### Clonogenic forming assay

The medium of the cell cultures after treatment with the tested compounds and/or irradiation under aerobic or hypoxic conditions were first separated to remove the tested compounds, and washed with PBS. Cells were seeded in 6-well plates at concentration of 5 × 10^3^ cells per well and maintained at 37°C for 14 days. The cells were then fixed in methanol, colonies which were formed stained with crystal violet and those containing more than 50 cells were scored. The number of colonies of treated and untreated dishes with the tested compounds were compared and expressed as the percentage of survival by clonogenic assay [18]. Clonogenic survival curves were constructed by plotting percent of survivals in response to different concentrations of the tested compounds.

### Radiosensitizing activity

#### Irradiation

HT-29 cells at 37°C under hypoxic or aerobic conditions were incubated with the tested compounds at concentrations of 25, 50, and 100 μmol for 3 h and then at room temperature were exposed to 4 different doses of radiation (2, 4, 8 and 12 Gy) at the dose rate of 1.67 Gy/min and Source Surface Distance (SSD) = 80 cm^2^ by a ^60^Co source (Theratron 780 MDS – Nordion).

#### Radiosensitivity measurement

Radiosensitivity of the tested compounds were determined through measurement of the cell growth inhibition using clonogenic assay [18]. Results are presented in terms of Sensitization Enhancement Ratio (SER) which were calculated from the equation SER = Do untreated cells/ Do treated cells, where Do values represent radiation dose that leads to 37% cell survival [20]. Do values were obtained from the survival curves of irradiation in the presence and absence of the tested compounds.

### Statistical analyses

For the cell survival and cell death assays each experiment was repeated three times and results are mean ± SD of three independent experiments. Statistical analysis was performed using Sigmaplot 12 (Systat Software Inc.) for window and descriptive statistics are shown as arithmetic mean ± standard deviation. Independent samples’ t-test was used to investigate the mean differences between irradiated and un-irradiated cells treated with the tested compounds and for comparison of more than two groups, Holm-Sidak multiple comparison test followed by one way analysis of variance (One way ANOVA) were performed and p value smaller than 0.05 was considered statistically significant.

## Results and discussion

**Scheme 1:**
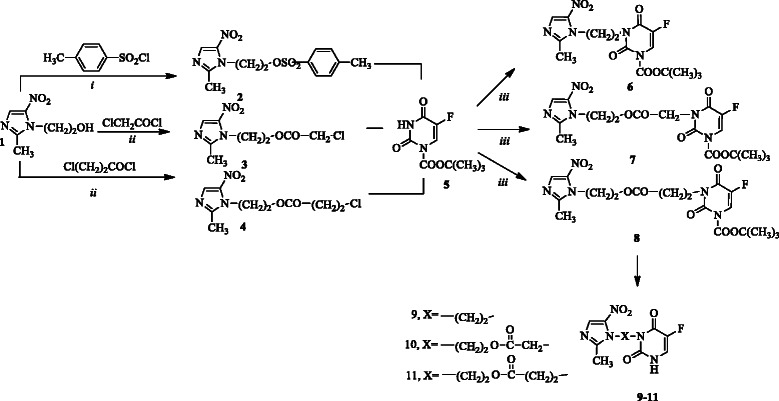
**Reagent and conditions for the synthesis of compounds 7–9.** (i) Pyridine <15°C, (ii) CH_2_Cl_2_, triethylamine, r. t, (iii) 4-DMAP/CH_3_CN, (iv) DMF, NaH, 80°C, (v) CH_3_OH, K_2_CO_3._

Tethered compounds **7**–**9** were prepared by the reaction of 1-(*t*-butoxycarbonyl)-5-FU **6**[13] with p-toluenesulfonic, 2-chloroacetic and 3-chloropropionic acid esters of metronidazole **1** (compounds **2–4**, Scheme 1) respectively in DMF in the presence of sodium hydride followed by removal of *t*-butoxycarbonyl group in methanolic potassium carbonate. Esters **3, 4** were obtained by the reported method for the preparation of the ester **2**[21] through the reaction of metronidazole **1** with 2-chloroacetyl chloride, and 3-chloropropionyl chloride respectively in pyridine.

**Figure 1:**
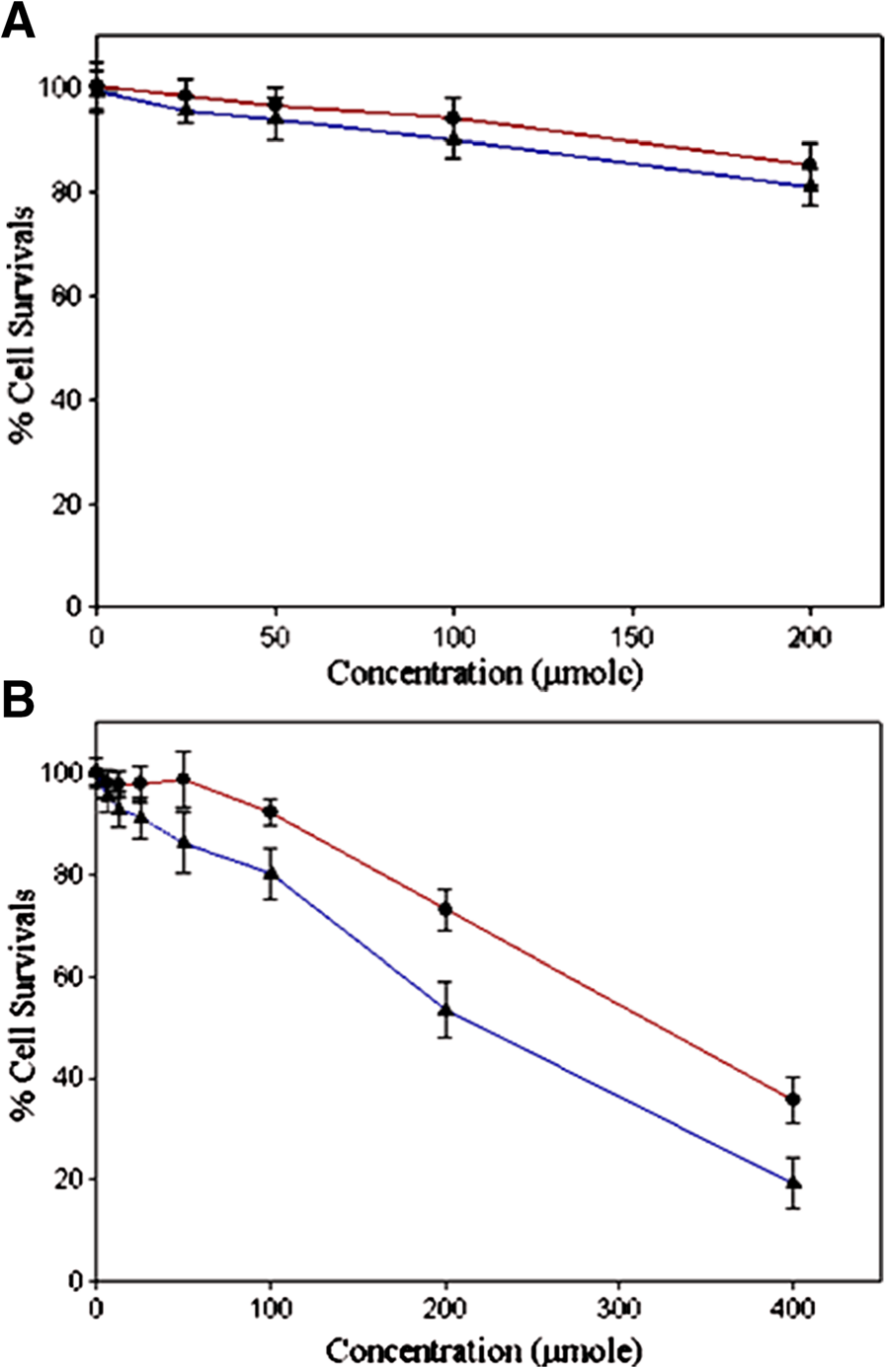
**Clonogenic survival of incubation of HT-29 cell line with different concentration of compound 7 after 3 h (A) and 48 h (B) under aerobic (•) and hypoxic (▲) conditions.** Data points were obtained from three independent experiments, and the standard deviations are given for each point. (Mean ± SD each set of experiments was performed in triplicates).

Effects of different concentrations of 5FU **5**, metronidazole **1**, and tethered compounds **7**–**9** for different incubation periods on viability and proliferation of HT-29 cells were determined by MTT (15), PI-digitonin [16,17], and clonogenic assays (18). The tested compounds inhibited HT-29 cell proliferation in a time dependent manner and as it is shown for the compound **7** (Figure 1A) cell survival at 3 h after incubation were similar (p > 0.05) but after 48 h were significantly different (p < 0.05). IC_50_ values were assessed from cell survival curves in which the percentage of cells that survived after 48 h incubation were plotted as a function of the concentration of tested compounds (Table 1). Results of this study showed that IC_50_ values of each compound for the cell survival under aerobic or hypoxic conditions determined either by MTT or by PI-digitonin assays are not different significantly (p > 0.05) from each other and results of these tests which are seldom unanimous [12] were in good agreement.

**Table 1:** Cytotoxicity and radiosensitization of metronidazole 1, 5-fluorouracil 5, and tethered compounds 7-9

	**IC**_**50 **_**(μmole)**				**SER**					
**Compound**	**MTT assay**^**a**^		**PI assay**^**b**^		**Concentration (μmole)**					
					**25**		**50**		**100**	
	**Aerobic**	**Hypoxic**	**Aerobic**	**Hypoxic**	**Aerobic**	**Hypoxic**	**Aerobic**	**Hypoxic**	**Aerobic**	**Hypoxic**
**1**	338 ± 12	265.9 ± 19	311 ± 16	250 ± 23	1.07 ± 0.06	1.26 ± 0.02	1.15 ± 0.09	1.69 ± 0.08	1.27 ± 0.02	2.06 ± 0.06
**5**	93 ± 9	89 ± 7	88 ± 7	84 ± 5	1.09 ± 0.06	1.04 ± 0.04	1.12 ± 0.09	1.18 ± 0.11	1.16 ± 0.11	1.12 ± 0.06
**7**	330 ± 16	240 ± 14	318 ± 20	230 ± 11	1.11 ± 0.11	1.41 ± 0.06	1.19 ± 0.05	1.85 ± 0.05	1.25 ± 0.010	2.17 ± 0.11
**8**	309 ± 25	232.1 ± 21	296 ± 18	229 ± 19	1.07 ± 0.03	1.51 ± 0.12	1.13 ± 0.08	1.95 ± 0.12	1.31 ± 0.12	2.26 ± 0.16
**9**	304 ± 18	234 ± 11	299 ± 14	224 ± 25	1.13 ± 0.06	1.43 ± 0.07	1.22 ± 0.10	1.79 ± 0.05	1.33 ± 0.05	2.13 ± 0.08

^a^3-(4,5-Dimethylthiazol-2-yl)-2,5-diphenyltetrazolium bromide.

^b^Propidium Iodide-Digitonin assay.

While tethered compounds **7**–**9** under hypoxic conditions exhibited remarkable concentration–dependent cytotoxicity (Figure 1B), these compounds under aerobic conditions were not or were slightly cytotoxic whereas at concentrations up to 100 μmol could reduce viabilities of HT-29 cells only to less than 10% (Figure 1B). Consistent with these results the IC_50_ values of these compounds were significantly lower (p <0.05) under hypoxic (232.1±21-240±14) compared to aerobic (304±18-330±16) conditions suggesting that compounds have hypoxia-selective cytotoxicity.

**Figure 2:**
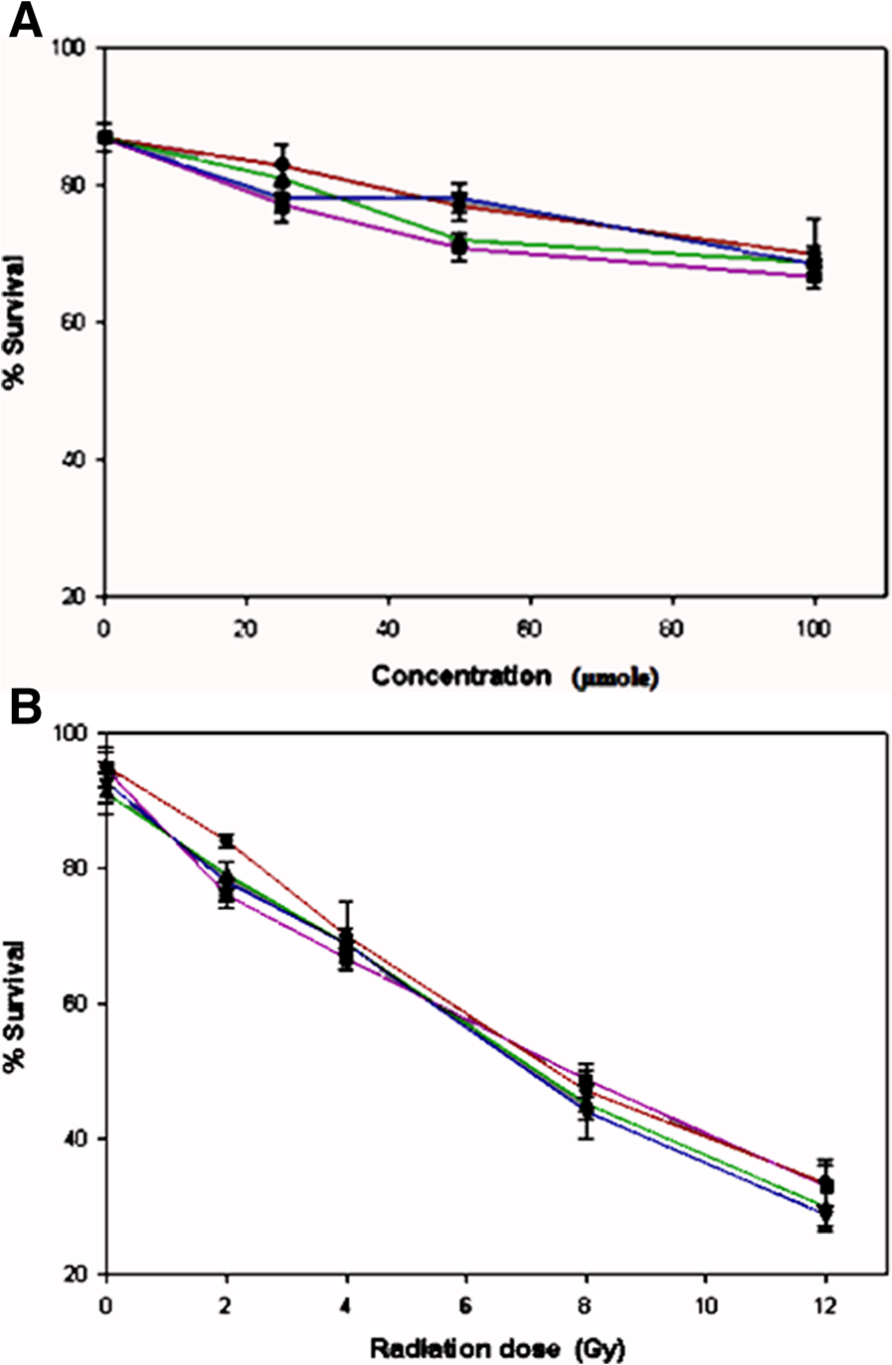
**Clonogenic cell survival of HT-29 cells under aerobic conditions, (A): upon exposure to 4 Gy gamma-irradiation after incubation with different concentration (25, 50, 100 μmole) of metronidazole 1 and tethered compounds 7–9 for 3 h, (B): upon exposure to different doses (2, 4, 8 and 12 Gy) of radiation after incubation with 50 μmol of metronidazole 1 and tethered compounds 7–9 for 3 h.** Each data points were obtained from three independent experiments, and the standard deviations are given for each point. (Mean ± SD).

**Figure 3:**
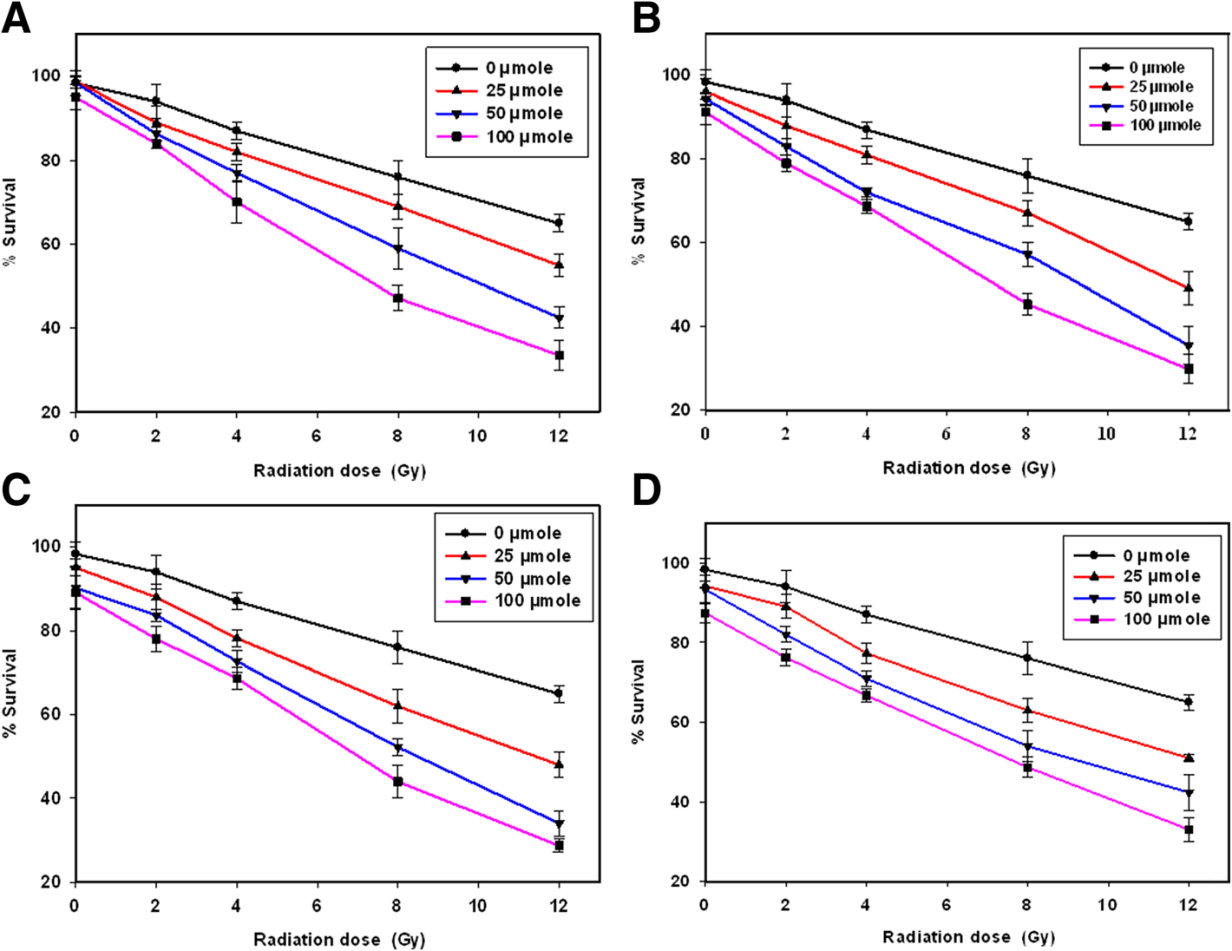
**Clonogenic cell survival of HT-29 cells upon exposure to different doses of Gamma-radiation (2, 4, 6, 8 and 12 Gy) after incubation for 3 h with different concentrations (0, 25, 50 and 100 μmole) of metronidazole 1 and tethered compounds 7–9 under hypoxic conditions.** Data points were obtained from three independent experiments, and the standard deviations are given for each point. (Mean ± SD, each set of experiments was performed in triplicates).

The radiosensitizing activities of all tested compounds were determined by measurement of the cell growth inhibition using a standard colony formation method following exposure of incubations with different concentrations of the tested compounds to different doses of the radiation [18]. Results are presented in term of Sensitization Enhancement Ratio (SER) which were obtained from the survival curves of irradiation in the presence and absence of the tested compounds for 37% survival respectively (20). The radiosensitization effects of compounds **7**–**9** at various concentrations under aerobic conditions were not different significantly (p > 0.05) (Figure 2A) and at a given concentration only increase in the dose of radiation resulted in decrease in the cell survival (Figure 2B). However under hypoxic conditions (Figures 3) radiosensitization effects of these compounds increased significantly with increase in their concentrations as well as the dose of radiation ( p < 0.05) These results were further supported by their SER values (Table 1) which under hypoxic conditions were concentration- dependent and relative to aerobic conditions were significantly higher (p < 0.05). The SER values of tethered compound **7**–**9** under aerobic conditions at all concentrations were lower and under hypoxic conditions at concentrations of 50 and 100 μmol were higher than 1.6 which is the minimum required value [22] for an *in vitro* radiosensitizing activity, indicating that these compounds have hypoxia-selective radiosensitization effects. From the results of this study it appears that the functional groups and the length of the linker chain have no contribution on *in vitro* effects of tethered compounds **7**–**9** since cytotoxities and radiosensitizing activities of these compounds under either hypoxic or aerobic conditions were not significantly different from each other.

## Conclusion

While the cytotoxicity of 5-FU **5** against HT-29 cell line under aerobic and hypoxic conditions were not different significantly and this compound at all concentrations showed no radiosensitizing activity, tethered compounds **7**–**9** showed higher cytotoxicities and selective radiosensitizing activities under hypoxic conditions. Also effects of these compounds relative to metronidazole **1** which is a known cytotoxin and radiosensitizer were comparable under aerobic but higher under hypoxic conditions. Further exploration of these compounds through modification of the linker chain might result in compounds which their uses in conjunction with tumor radiotherapy have greater toxicity to tumor than normal tissues.

## Abbreviations

5 FU: 5- fluorouracil; MTT: 3-(4,5-Dimethylthiazol-2-yl)-2,5-diphenyltetrazolium bromide; PI: Propidium Iodide; SER: Sensitization enhancement ratio; PBS: Phosphate buffer saline; FBS: Fetal bovine serum.

## Competing interests

The authors declare that they have no competing interests.

## Authors’ contributions

KA: Designer of the project, corresponding author of the manuscript. AK: Determination of cytotoxicity and radiosensitizing activity of the tested compounds. OS-N: Supervisor for the determination of the cytotoxocity and radiosensitizing activity of the tested compounds. LN: Synthesis of the tethered compounds. KMR: supervising statistical analyses. All authors read and approved the final manuscript.
